# Adolescents’ multiple, recurrent subjective health complaints: investigating associations with emotional/behavioural difficulties in a cross-sectional, school-based study

**DOI:** 10.1186/1753-2000-8-3

**Published:** 2014-01-24

**Authors:** Dimitra Petanidou, George Giannakopoulos, Chara Tzavara, Christine Dimitrakaki, Gerasimos Kolaitis, Yannis Tountas

**Affiliations:** 1 Centre for Health Services Research, Department of Hygiene, Epidemiology and Medical Statistics, Athens University Medical School, 25 Alexandroupoleos str., 11527 Athens, Greece; 2 Department of Child Psychiatry, Athens University Medical School, “Aghia Sophia” Children’s Hospital, Greece, Thivon and Papadiamantopoulou, 115 27 Athens, Greece

**Keywords:** Subjective Health Complaints (SHC), Emotional/behavioural difficulties, SDQ scale, Adolescents

## Abstract

**Background:**

Adolescence has been documented as the peak age of onset for mental health perturbations, clinical disorders and unsubstantiated health complaints. The present study attempted to investigate associations between multiple, recurrent subjective health complaints (SHC) with emotional/behavioural difficulties, as measured by the Strengths and Difficulties Questionnaire scale (SDQ), among Greek adolescents.

**Methods:**

Questionnaires were administered in a large, nation-wide, random, school-based sample of Greek adolescents, aged 12–18 years. Data from 1170 participants were analyzed. Adolescents with multiple, recurrent SHC were compared in terms of their emotional/behavioural difficulties to their peers with lower levels of health complaints. SDQ scales were separately investigated for their associations with multiple, recurrent SHC, after adjustment for gender, age and socioeconomic status (ses). Further analysis included multiple logistic regression models with multiple, recurrent SHC as the dependent variable and gender, age, ses and SDQ Total difficulties score as independent factors. Potential gender and age interactions were also explored.

**Results:**

Almost half of the study participants reported multiple, recurrent SHC. Adolescents with multiple, recurrent SHC had higher scores on all SDQ scales, except from the Prosocial behavior scale, compared to their peers with lower levels of health complaints. Emotional Symptoms, Conduct Problems, Hyperactivity/Inattention and Peer Problems were associated with greater likelihood of having multiple, recurrent SHC, after adjustment for gender, age and ses. The multiple logistic regression models revealed that older adolescents and girls, as well as those with increased Total difficulties score had an increased risk for multiple, recurrent SHC reporting. No significant interaction between SDQ scales and gender or age was found.

**Conclusions:**

Our study highlights the magnitude of psychological burden among adolescents experiencing multiple, recurrent SHC. Professionals in school and clinical settings should be cautious for impaired emotional/behavioural functioning when assessing adolescents with multiple, recurrent SHC, so as early identification of at-risk individuals and timely, appropriate referrals are facilitated.

## Background

Even though adolescence is typically viewed as a period of good physical health, it has also been established as the peak age of onset for mental health perturbations and clinical disorders [1,2]. Symptoms of emotional distress, behavioural difficulties, introspectiveness and health complaints unattributed to a clear medical or psychological diagnosis – such as headaches, irritability and nervousness, broadly labeled as ’Subjective Health Complaints” (SHC)– have been commonly considered transient, accompanying features of the developmental course to adulthood. Conversely, these symptoms may be of sufficient number and severity to constitute a significant public health issue across childhood and adolescence. As reported from international studies, prevalence estimates for emotional and conduct disorders range from 10-20% [3], while an average of 28-35% of schoolchildren aged 11–18 years report multiple (two or more) SHC at least once per week across 39 countries [4].

Although emotional and behavioural problems are highly prevalent internationally, they remain largely undetected, as children and adolescents in need scarcely reach appropriate mental health consultation services [1]. On the other hand, SHC are one of the main reasons for paediatric primary care visits and a frustrating puzzle for health professionals, who strain to treat vague and unsubstantiated symptoms that cause physical and/or psychological distress [5]. They often turn to thorough –and, sometimes, costly– medical examinations and fruitless interventions, but rarely proceed with or refer for a generic mental health assessment, therefore contributing to an incomplete and fragmentary treatment of the affected individual [6,7].

However, a large body of evidence ascertains that SHC are significantly related to depressive and anxiety symptoms as well as to the full-blown, respective clinical syndromes [8]. Research on paediatric community samples has shown that older children and adolescents with multiple, recurrent health complaints [9-13] –mainly headache and abdominal pains [14], as well as musculoskeletal symptoms [15] and fatigue [16]– have an amplified risk to experience anxiety and depressive symptoms. The above outcomes have been corroborated by longitudinal research evidence [5,17-23], even though not consistently [12,23-25]. Regarding the associations of SHC with externalizing symptoms and disorders, such as hyperactivity/inattention, conduct problems and difficulties in social interactions, research so far has been less extensive and less conclusive in its findings. Whereas some studies have found that levels of behavioural problems did not differ between somatizing and non-somatizing children [6,25], one study has reported lower levels for somatizers [7], while a respectable amount of studies have supported a link between behavioural difficulties and SHC and pain symptoms [10,19,21,26], headaches [27,28], abdominal [29,30] and musculoskeletal pain [31,32]. Hyperactivity/inattention difficulties and peer problems have also been positively associated with SHC and pain symptoms, especially headache [10,26-28,33].

Even though methodological differences and limitations of existing studies hamper the possibility to determine the extent to which SHC and emotional/behavioural difficulties are reciprocally predictive of one another [34], a “dose–response” relationship has been suggested. Increasing numbers of SHC have been associated with higher levels of anxiety and depressive symptoms, as well as of externalizing symptoms, signaling a shift of research focus from specific types of symptoms to the number and frequency of co-occurring symptoms [12,21,23,35]. Against this background, presentations of multiple, recurrent SHC in paediatric primary care services could foster the early identification of individuals with an elevated risk for emotional/behavioural problems and, thus, represent a viable window for timely mental health interventions.

With respect to other significant factors in the interplay between emotional/behavioural problems and disorders and paediatric SHC, gender has been consistently highlighted for its salient effect. Apart from a well-documented female predisposition for increased reports of psychosomatic ailments [10,13,36], SHC and various pain symptoms have been associated with emotional difficulties in girls and externalizing symptoms in boys [20,21,37]. On the other hand, the effect of age on the association of SHC with emotional/behavioural difficulties remains ambiguous, with some evidence disclaiming an age interaction on the aforementioned relationship [12,33,38].

Based on the scarcity of pertinent research in Greece [39], as well as on existing inconclusive research evidence, the aim of the present study was three-fold: a) to elucidate potential differences in the emotional/behavioural functioning of adolescents who report multiple, recurrent SHC compared to peers with less – in terms of both number and frequency– SHC in a nation-wide, random, school-based sample, b) to investigate the associations of adolescents’ emotional and behavioural difficulties as measured by the SDQ scale with multiple, recurrent SHC after adjusting for the effects of gender, age and family socio-economic status (ses) and c) to explore for gender and age effects on the aforementioned associations. We expected that higher scores on all SDQ scales (except from pro-social behaviour) would correspond to those adolescents who reported multiple, recurrent SHC. Another hypothesis was that adolescents with emotional/behavioural difficulties, as measured by the SDQ scale, would be at increased risk for multiple, recurrent SHC reporting, after adjustment for gender, age and ses. Also, we speculated that the associations between SDQ scales and SHC would be different across gender and age groups.

## Methods

### Participants and procedure

The study was conducted in 2003 within the framework of the European project “Screening and Promotion for Health Related Quality of Life (HRQoL) in Children and Adolescents: A European Public Health Perspective” (acronym: KIDSCREEN) [40]. The school sampling in Greece was random, multi-staged and based on the age and gender distribution of school children living in the 54 geographical sectors of the country, according to data from the National Census of 2001. Schools in each sector were randomly selected by a computer program and students of each selected school were selected randomly from classroom name lists. Ethical approval was attained from the National Ministry of Education. A sample of 1900 adolescents (12 to 18 year olds) was recruited. The KIDSCREEN questionnaires were accompanied by the parents’ information letter, an informed consent form, and the information letter for the students. The consent to participate was obtained before survey administration. Inclusion criteria for students were: to belong in the age group under study, to be able to read and complete the questionnaire themselves and to consent to take part in the study. Students completed the questionnaire at school. A total of 1194 (i.e. 63% response rate) of self-reported questionnaires were finally returned. Data from 1170 adolescents were analyzed. Previous research on the representativeness of the present sample has reported that non-responder interviews showed no significant differences between responders and non-responders with regard to adolescents’ and parents’ general perceived health, parents’ marital status and highest educational level, and type of residence, indicating that a selection bias is less likely [41].

### Measures

#### Subjective health complaints (SHC)

SHC were measured through the Health Behaviour in School-aged Children Symptom Checklist (HBSC-SCL; [42]), a self-administered brief screening instrument which indicates the frequency of occurrence of eight common health complaints. Students were asked: “In the last 6 months how often have you had the following?” and the items included were: headache, stomachache, backache, depressed mood, irritability, nervousness, sleeping difficulties, dizziness. Each health complaint was rated on a five-point frequency scale: “about every day” (5), “more than once a week” (4), “about every week” (3), “about every month” (2) and “rarely/never” (1). Following previous, relevant research stressing the co-occurrence of recurrent health complaints [12,36,43], we considered that the presence of two or more SHC more than once a week could reflect a noticeable impairment in adolescents’ psychosomatic adjustment. Therefore, a dichotomous variable was created, according to which adolescents who reported at least two SHC more than once a week – corresponding to scoring categories 4 and 5– were categorized in the “Multiple Recurrent SHC” group (MR-SHC). Adolescents who reported less frequent and fewer SHC –corresponding to scoring categories 2 and 3– were grouped together with those who reported rare or no experiences of SHC (scoring category 1) and formed the “no Multiple-Recurrent SHC” group (no MR-SHC). In quantitative analysis the HBSC-SCL items have revealed adequate validity and reliability properties [44]. The Cronbach's α coefficient in the present study was found to be acceptable (α = 0.79).

#### Emotional/behavioural problems

To assess adolescents’ emotional/behavioural problems, the Strengths and Difficulties Questionnaire (SDQ; [45]) was used. The SDQ contains 25 items (small sentences), categorized into five scales of five items each: hyperactivity/inattention, emotional symptoms, conduct problems, peer problems and prosocial behaviour. Responses to each of the 25 items consisted of three options: not true, somewhat true, or certainly true. For all scales the items that are worded negatively are assigned scores of 2 for certainly true, 1 for somewhat true, and 0 for not true. All but the last scale can be summed up to a total difficulties score ranging from 0 to 40. Before the statistical analysis, the item concerning somatic symptoms in the emotional symptoms scale was excluded. In order to combat inherent weaknesses of cross-cultural adaptation (e.g., semantic and scale equivalence) the research team followed a standardized translation methodology according to international cross-cultural translation guidelines [46]. In the present study, the Cronbach α coefficient for SDQ total difficulties score was 0.79 and it ranged from 0.50 to 0.71 for the individual scales, in accordance with previous Greek psychometric studies [47,48]. The version for youths was used in the present study.

#### Socio-economic status (SES)

SES was measured by the Family Affluence Scale (FAS; [49]), an indicator of family wealth addressed to child and adolescent populations that includes family car ownership, having their own unshared bedroom, the number of computers at home and times the child spent on holidays in the past 12 months. FAS is usually collected in 8 categories (from 0, the lowest, to 7, the highest FAS category). In the present study it was re-coded into 3 groups in the analysis (low FAS level [0–3], and medium [4,5] and high FAS level [6,7]). FAS exhibits acceptable psychometric properties and has been commonly used in relevant studies [36,42].

#### Gender-age

Gender was identified based upon the survey responses to the question “Are you a boy or a girl?” Age was calculated by subtracting the date of birth from the interview date and was classified according to date of birth in two categories: 12 to 15 years and 16 to 18 years.

### Statistical analysis

Continuous variables are presented with mean and standard deviation while quantitative variables are presented with absolute and relative frequencies. Student’s t-tests were computed for the comparison of mean SDQ scale values between the “no MR-SHC” and the “MR-SHC” group. Data were further analysed using multiple logistic regression analysis with dependent the variable presented if the adolescents had multiple, recurrent SHC and independent variables the SDQ scales, gender, age and socioeconomic status. Each SDQ scale was examined separately in the logistic regression model because model diagnostics with two or more SDQ scales in the models indicated that the regression estimates were highly collinear. Adjusted odds ratios with 95% confidence intervals were computed from the results of the logistic regression analyses. Model diagnostics were evaluated using the Hosmer and Lemeshow statistic. Hypothesized interactions of variables in the models were not significant. All p values reported are two-tailed. Statistical significance was set at 0.05 and analyses were conducted using SPSS statistical software (version 19.0).

## Results

Data from 1170 participants with information about multiple, recurrent SHC (468 males and 702 females) were analysed. Sample characteristics are presented in Table 1. Almost half of the adolescents (45.8%) were categorized as having multiple, recurrent SHC (“MR-SHC” group). The rest reported lower levels of SHC and were coded as “no MR-SHC” group. Mean SDQ scales for the no MR-SHC and MR-SHC groups are shown in Table 2. Adolescents of the MR-SHC group had greater scores on all SDQ subscales except for Prosocial Behaviour compared to those belonging to the no MR-SHC group. When multiple logistic regression analysis was conducted with multiple, recurrent SHC as the dependent variable and SDQ subscales scores as the independent variables and after adjusting for gender, age and FAS (Table 3) it was found that increased scores on Emotional Symptoms, Conduct Problems, Hyperactivity/Inattention and Peer Problems were associated with greater likelihood for having multiple, recurrent SHC. Results of multiple logistic regression model with independent variable the total SDQ score are shown in Table 4. Increased Total difficulties score was associated with greater odds for having multiple, recurrent SHC (0R = 1.23, 95% CI: 1.18-1.27), while the likelihood of having multiple, recurrent SHC was greater in girls and adolescents aged 16 to 18 years compared to those aged 12 to 15 years. No significant interaction between SDQ scales and gender or age was found indicating that the effect of Emotional Symptoms, Conduct Problems, Hyperactivity, Peer Problems and total difficulties on having multiple, recurrent SHC was similar between girls and boys or between younger and older adolescents.

**Table 1:** Sample characteristics

	**N (%)**
Gender	
Girls	702 (60.0)
Boys	468 (40.0)
Age (years)	
12-15	794 (67.9)
16-18	376 (32.1)
Family affluence scale	
Low	412 (37.7)
Medium	489 (44.7)
High	192 (17.6)
SHC Group	
no MR-SHC^‡^	634 (54.2)
MR-SHC^†^	536 (45.8)
SDQ *scales*	
Emotional symptoms, mean (SD)	2.7 (1.9)
Conduct problems, mean (SD)	3.0 (1.5)
Hyperactivity/Inattention, mean (SD)	3.6 (2.2)
Peer problems, mean (SD)	1.9 (1.7)
Prosocial behaviour, mean (SD)	8.1 (1.9)
Total difficulties, mean (SD)	11.1 (5.2)

^‡^Adolescents with low levels of SHC.

^†^Adolescents with multiple, recurrent SHC.

**Table 2:** Mean SDQ scales scores for adolescents in the MHC and no-MHC group

**SDQ scales**	**No MR-SHC**^**‡**^	**MR-SHC**^**†**^	**P**
	**N (%)**	**N (%)**	
Emotional symptoms	2.1 (1.7)	3.5 (1.9)	<0.001
Conduct problems	2.5 (1.4)	3.5 (1.6)	<0.001
Hyperactivity/Inattention	2.9 (2.0)	4.4 (2.1)	<0.001
Peer problems	1.6 (1.6)	2.2 (1.8)	<0.001
Prosocial behaviour	8.1 (1.8)	7.9 (1.9)	0.087
Total difficulties	9.0 (4.7)	13.5 (4.8)	<0.001

^‡^Adolescents with low levels of SHC.

^†^MR-SHC group: adolescents with multiple, recurrent SHC.

**Table 3:** Odds ratios (95% confidence intervals) of multiple, recurrent SHC in association with SDQ scales scores

	**OR (95% CI)**^**‡**^	**P**
Emotional symptoms	1.54 (1.43 – 1.67)	<0.001
Conduct problems	1.62 (1.47 – 1.78)	<0.001
Hyperactivity/Inattention	1.40 (1.31 – 1.5)	<0.001
Peer problems	1.27 (1.17 – 1.37)	<0.001
Prosocial behaviour	0.94 (0.88 – 1.01)	0.087

^‡^adjusted for gender, age and FAS.

**Table 4:** Odds ratios (95% confidence intervals) of multiple, recurrent SHC in association with SDQ total score

	**OR (95% CI)**	**P**
Gender		
Girls	1.00*	
Boys	1.47 (1.09 – 1.99)	0.013
Age (years)		
12-15	1.00*	
16-18	1.48 (1.13 – 1.94)	0.005
Family affluence scale (low medium high FAS)		
Low	1.00*	
Medium	0.95 (0.71 – 1.25)	0.701
High	1.21 (0.74 – 1.86)	0.302
Total difficulties (SDQ)	1.23 (1.18 – 1.27)	<0.001

*indicates reference category.

## Discussion

The main purpose of the present study was to cast light upon associations between impaired emotional and behavioural functioning and SHC in a large, national, school-based adolescent sample. Building on previous research emphasizing the frequency of co-occurring symptoms, rather than specific conditions, we focused on multiple, recurrent SHC as they have been suggested to be indicative of remarkable psychosomatic disturbance [36,43].

Almost half of the study participants reported more than two SHC on a weekly basis. Previous research has postulated that clustering of co-occurring psychosomatic symptoms is common among adolescents, especially in the Southern regions of Europe [4,36,43]. Moreover, our analysis showed that adolescents with multiple, recurrent SHC had higher levels of emotional/behavioural problems, revealing a significantly impaired psychosocial functioning, in comparison with peers with low levels of SHC. Poor mental health outcomes have been previously documented among community adolescent and clinical paediatric samples who reported multiple sites of pain [9,26] and frequent co-occurring pain symptoms [6,50].

In line with previous evidence regarding female and older adolescents’ predominance in SHC [4,13,36,51], the present study showed that multiple, recurrent SHC were more likely to be reported by girls and older adolescents, as well as by their counterparts with higher levels of emotional/behavioural problems. Additionally, adolescents’ socio-economic background was not proved to be significantly associated with multiple, recurrent SHC. Although there is plenty of evidence running counter to this finding [4,36,52], a previous study employing the same sample showed that family socioeconomic status had only a minor effect on self-reported SHC [51]; instead, it has been suggested that the subjective perception of one’s financial resources in relation to his/her peers may constitute a more sensitive predictor of health during adolescent years [51,53].

Focusing on the relationships between SHC and emotional/behavioural problems, further analysis revealed significant, albeit moderate to weak, associations. Specifically, after adjustment for confounding factors, the highest odds for multiple, recurrent symptom reporting was shown for adolescents with conduct problems, followed by those with emotional symptoms, hyperactivity/inattention difficulties and peer problems. Apart from supporting the well-established relation between SHC and emotional symptoms [34], our study documented an elevated risk of multiple, recurrent SHC in adolescents with conduct problems, adding further support to existing non-conclusive findings [10,12,27,28]. Moreover, the significant relationship of SHC with hyperactivity/inattention and peer problems reported here agrees with findings from research on specific symptoms and chronic pain [10,27,31,33].

Efforts to explain the link between SHC and impaired emotional/behavioural functioning have mainly focused on depression and anxiety symptoms. They include unidirectional causal models, where one constellation of symptoms causes or increases the risk for the other, and shared vulnerability models, where comorbid symptoms share common risk factors or serve as different facets of the same underlying process [34]. When it comes to behavioural problems, respective research is still meager. A well-grounded pathway that may explain the link between SHC and adjustment problems in adolescence lies on Pulkinnen’s model of emotional and behavioural regulation. According to his model, the mechanisms of emotion and behavioural regulation help to maintain internal arousal within a manageable performance range and to adjust behavioural expression to external circumstances. In this framework, externalizing problems, that are characterized by intense emotions and active behavior, may relate to perceptions of pain in a bidirectional way: pain experiences or vague disturbances may act as stressors that augment the level of emotional distress, which, in turn, lowers the threshold for pain perception; conversely, emotional distress per se (eg. aggression, negative affectivity) may attenuate individual’s coping capacity resulting in the intensification of the stress experience, that, in turn, exacerbates pain perception. Therefore, multiple, recurrent SHC may constitute behavioural manifestations that depict the low self-control capacity on managing intensification of emotions and activation of behaviour [54]. What is more, neurobiological findings regarding the close proximity of brain structures processing pain and negative emotion may provide impetus to study the association of emotional/behavioural functioning and SHC from a neuropsychological perspective as well [20].

Contrary to our expectations, we did not find any significant interactions between gender or age and emotional/behavioural difficulties on self-reported multiple, recurrent SHC. Nonetheless, specific somatic complaints (stomachaches, musculoskeletal pains and headaches) have been found to associate strongly with emotional disorders in girls and with disruptive behaviour disorders in boys [37]. In the same line, a female predisposition to internalizing symptoms and a male tendency to externalizing problems have been reported among adolescents experiencing two or more sites of recurrent pain [20,21]. What is more, gender and age differences have been documented for emotional/behavioural difficulties, as measured by SDQ, during adolescent years [47,48].

Yet, our finding suggests that the effect of emotional symptoms, conduct problems, hyperactivity/inattention, peer problems and total difficulties on the odds for multiple, recurrent SHC was similar between boys and girls as well as between younger and older adolescents. Therefore, it could be inferred that gender, age and emotional/behavioural difficulties have significant, additive effects on multiple, recurrent SHC. Similarly, Tangen et al. [38] showed that the association of anxiety and depression with functional somatic symptoms was equally strong across gender and age groups in a large, adult population study. Lack of significant gender or age interactions were also reported by Dhossche et al. [12] when examining the robust association between number of functional somatic symptoms and depressive and anxiety disorders among young adults. In this longitudinal study [12], they demonstrated that there was no significant age effect on the association between adolescents’ functional somatic symptoms and externalizing problems. In the same line, Vaalamo et al. [54] showed no differences in externalizing problem behaviours between 11–12 years old boys and girls with recurrent pain.

However, the abovementioned inconsistent research evidence could be attributed, at least in part, to social and cross-cultural differences among populations under study, especially with respect to gender role expectations and gender normative behaviour, even in the face of mental health distress manifestations. In addition, it could be speculated that other factors, not measured in the present study, that have been previously identified as salient determinants of both emotional/behavioural functioning and SHC in adolescence, could contribute in a more thorough understanding of the relationship between adolescents’ self-reported SHC and their emotional/behavioural problems. These include adolescent-specific characteristics like temperamental traits as well as other features of adolescents’ lives such as the quality of parent–child relationship, parental mental health status and exposure to adverse life events [34]. Therefore, we would encourage future research to elaborate on the relationship of adolescents’ emotional/behavioural difficulties with SHC by employing more pertinent factors and by exploring for potential gender or age interactions across multiple contexts.

Our study is one of the few to focus on the association of emotional/behavioural symptoms, as measured by the SDQ scale, with multiple, recurrent SHC, extending, thus, previous relevant research on isolated pain and functional somatic symptoms to a wider psychosomatic frame of distress. In addition, the large, nation-wide, random, school-based sample has been one of the major strengths of the present study. It should be also stressed that although the tools employed do not indicate specific psychiatric diagnosis, they have been standardized and widely used among adolescent samples for screening purposes [47,48].

In discussing study limitations, it should be acknowledged that there was a tendency in our sample for a higher response rate from girls compared with boys and from younger participants in relation to older ones. Even though this tendency is commonly met in school-based surveys and across sampling methods and countries, caution is required since school-based surveys do not necessarily provide the most representative samples, at least in terms of age and gender. However, in the context of the KIDSCREEN study, further procedures were implemented in order to remove bias in the sample due to nonresponse and non-coverage errors, as well as to assess the representativeness of national samples. These showed that the KIDSCREEN survey is sufficiently representative when it comes to providing reference population values [41]. In addition, the cross-sectional study design indicates a complex, bidirectional relationship between emotional/behavioural difficulties and multiple, recurrent SHC, but it does not allow us to make causal inferences. Finally, it should be stressed that our findings may – to some extent– depict biased estimates because of their over-reliance on self-reported data. In addition, SHC reports could reflect a recall bias, as a result of their retrospective evaluation within a 6 months’ time frame.

## Conclusions

Our findings add support to the growing literature underlining the magnitude of psychological burden among adolescents experiencing multiple, recurrent SHC [9,13,55]. Psychosomatic disturbances, emotional symptoms and behavioural difficulties seem to be in a vicious circle of distress that hampers adolescents’ psychosocial adjustment and hinders present and future achievements. Adolescents’ reports of multiple, recurrent health complaints may be indicative of an underlying vulnerability to emotional symptoms and behavioural difficulties, and mainly conduct, depression and anxiety, hyperactivity/inattention and peer problems. Given that adolescence is considered as a key developmental period for the onset of major psychiatric disorders [2], health professionals in school and clinical settings are faced with a unique challenge, when evaluating adolescents who frequently present with multiple SHC. Assessing the potential underlying mental health difficulties of multiple, recurrent SHC could flag individuals in the process of transitioning from mental health vulnerability to being symptomatic [55]. Therefore, they need to be attentive to adolescents’ emotional/behavioural functioning and properly prepared to make appropriate referrals, when needed. Identification of the earliest phenotypes of mental health distress, a fundamental pillar of pre-emptive psychiatry, could lead to timely interventions and, in turn, to improved mental health outcomes in adolescent and adult years [2].

## Abbreviations

SHC: Subjective health complaints; SDQ: Strengths and difficulties questionnaire; SES: Socio-economic status; HRQoL: Health related quality of life; HBSC-SCL: Health behaviour in school-aged children - symptom check list; MR-SHC: Multiple, recurrent subjective health complaints; no MR-SHC: No-multiple, recurrent subjective health complaints; FAS: Family affluence scale.

## Competing interests

The authors declare that they have no competing interests.

## Authors’ contributions

DP carried out the writing of the manuscript. GG was involved in drafting and revising the manuscript. CC performed the statistical analysis. CD and GK participated in revising the paper. YT had overall supervision of the study. All authors read and approved the final manuscript.
